# TAX1BP1 contributes to deoxypodophyllotoxin-induced glioma cell parthanatos via inducing nuclear translocation of AIF by activation of mitochondrial respiratory chain complex I

**DOI:** 10.1038/s41401-023-01091-w

**Published:** 2023-04-25

**Authors:** Xuan-zhong Wang, Shi-peng Liang, Xi Chen, Zhen-chuan Wang, Chen Li, Chun-sheng Feng, Shan Lu, Chuan He, Yu-bo Wang, Guang-fan Chi, Peng-fei Ge

**Affiliations:** 1 Department of Neurosurgery, First Hospital of Jilin University, Changchun, 130021 China; 2 Research Center of Neuroscience, First Hospital of Jilin University, Changchun, 130021 China; 3 Department of Anesthesiology, First Hospital of Jilin University, Changchun, 130021 China; 4 Key Laboratory of Pathobiology, Ministry of Education, Jilin University, Changchun, 130021 China

**Keywords:** glioma, deoxypodophyllotoxin, parthanatos, apoptosis inducing factor (AIF), TAX1BP1, mitochondrial respiratory chain complex I

## Abstract

Parthanatos is a type of programmed cell death initiated by over-activated poly (ADP-ribose) polymerase 1 (PARP1). Nuclear translocation of apoptosis inducing factor (AIF) is a prominent feature of parthanatos. But it remains unclear how activated nuclear PARP1 induces mitochondrial AIF translocation into nuclei. Evidence has shown that deoxypodophyllotoxin (DPT) induces parthanatos in glioma cells via induction of excessive ROS. In this study we explored the downstream signal of activated PARP1 to induce nuclear translocation of AIF in DPT-triggered glioma cell parthanatos. We showed that treatment with DPT (450 nM) induced PARP1 over-activation and Tax1 binding protein 1 (TAX1BP1) distribution to mitochondria in human U87, U251 and U118 glioma cells. PARP1 activation promoted TAX1BP1 distribution to mitochondria by depleting nicotinamide adenine dinucleotide (NAD^+^). Knockdown of TAX1BP1 with siRNA not only inhibited TAX1BP1 accumulation in mitochondria, but also alleviated nuclear translocation of AIF and glioma cell death. We demonstrated that TAX1BP1 enhanced the activity of respiratory chain complex I not only by upregulating the expression of ND1, ND2, NDUFS2 and NDUFS4, but also promoting their assemblies into complex I. The activated respiratory complex I generated more superoxide to cause mitochondrial depolarization and nuclear translocation of AIF, while the increased mitochondrial superoxide reversely reinforced PARP1 activation by inducing ROS-dependent DNA double strand breaks. In mice bearing human U87 tumor xenograft, administration of DPT (10 mg· kg^−1^ ·d^−1^, i.p., for 8 days) markedly inhibited the tumor growth accompanied by NAD^+^ depletion, TAX1BP1 distribution to mitochondria, AIF distribution to nuclei as well as DNA DSBs and PARP1 activation in tumor tissues. Taken together, these data suggest that TAX1BP1 acts as a downstream signal of activated PARP1 to trigger nuclear translocation of AIF by activation of mitochondrial respiratory chain complex I.

## Introduction

Parthanatos is a type of non-apoptotic programmed cell death initiated by over-activated PARP1 and characterized by apoptosis inducing factor (AIF)-dependent DNA degradation [1]. After being activated upon occurrence of DNA double strand breaks (DSBs), nuclear PARP1 utilizes nicotinamide adenine dinucleotide (NAD^+^) as substrate to synthesize cytotoxic PAR polymers, which then translocate into cytoplasm and target mitochondria to induce AIF translocation from mitochondrial to nuclei [2]. Within nuclei, AIF acts as a nuclease to cause irreversible DNA degradation and cell death after being recruited to the location of DSBs [3]. Thus, nuclear translocation of AIF is a key step to complete the final stage of parthanatos. Although PAR polymers are regarded as the executioner to cause nuclear translocation of AIF during the process of parthanatos [4, 5], it remains unclear whether activated PARP1 induces AIF translocation from mitochondria to nuclei via regulation of other signals.

Mitochondrial respiratory chain complex I (NADH: ubiquinone oxidoreductase) is a protein complex responsible for acquiring electrons by oxidizing NADH to NAD^+^ and transferring these electrons to CoQ [6]. It is also a primary site where electrons leak into mitochondrial matrix and bind with molecular oxygen to form superoxide [6]. Given that mitochondrial superoxide is a crucial factor to cause nuclear translocation of AIF [7], activated complex I might play a role in promoting AIF translocation from mitochondria to nuclei. TAX1BP1 (Tax1 binding protein 1) is a protein highly and specifically expressed in brain and has dual roles in modulating the destinies of cancer cells [8]. On one hand, it inhibits the genesis and development of hepatocarcinoma [9]. On the other hand, it contributes to gastric cancer cells’ resistance to chemotherapeutic agent 5-FU [10]. TAX1BP1 was found to localize at mitochondria with decreased membrane potentials and accumulated superoxide [11], but it is unclear of its role in regulating mitochondrial respiratory complex I activity.

High-grade glioma is one of the leading causes of cancer mortality in adults and imposes huge challenges on its treatment [12]. Although chemotherapy based on inducing glioma cell apoptosis could prolong the survival of the patients with glioma to a certain extent, the prognosis is still poor due to inherent or acquired resistance [13]. Thus, developing new drugs to activate non-apoptotic cell death pathways is of great significance to treat glioma. Deoxypodophyllotoxin (DPT) is not only a natural lignan with multiple pharmacological effects such as anti-inflammation, antivirus and anti-allergy, but also could induce parthanatos in glioma cells [14]. However, the underlying mechanism accounting for its induction of parthanatos is still needed to be clarified. Therefore, we used DPT in this study to trigger glioma cell parthanatos and investigated whether TAX1BP1 is a downstream signal of activated PARP1 and initiates nuclear translocation of AIF via activation of respiratory complex I.

## Materials and methods

### Reagents

Deoxypodophyllotoxin (DPT) was obtained from Yuanye Bio-technology company (Shanghai, China). It was dissolved in DMSO and then stored at the concentration of 10 mmol/L. MnTBAP was from Sigma (St. Louis, MO, USA) and 3-Aminobenzamide (3AB), NAD^+^ and FK866 were all from MedChemExpress Company (Shanghai, China). The antibodies against TAX1BP1 (bs-13671R), NDUFS2 (bs-10455R) and NDUFS4 (bs-3961R) were all obtained from Bioss Antibodies (Beijing, China), against PARP1 (13371-1-AP), ND2 (19704-1-AP) and ND1 (19703-1-AP) were all from Proteintech Company (LA, USA), and against AIF (ab32516), GPX4 (ab125066), catalase (ab76024), H2AX (ab229914), TOMM20 (ab186735), phospho-H2AX at Ser139 (ab26350) and phospho-ATM at Ser1981 (ab81292) were all from Abcam company (Cambridge, UK), against PAR (#83732) and β-Actin (#4970) were both from Cell Signaling Technology Company (Beverly, MA, USA). Second antibody against rabbit (A0208) and against mouse (A0216) were from Beyotime Biotechnology (Nanjing, China).

### Cell line and culture

The human U87, U251 and U118 glioma cells obtained from Shanghai Institute of Cell Biology, Chinese Academy of Sciences were cultured at 37 °C in Dulbecco’s modified Eagle’s medium (DMEM) with high glucose, 10% fetal bovine serum, penicillin (100 U/mL) and streptomycin (100 μg/mL) in a humid environment containing 5% CO_2_.

### Assay of cell death ratio

The cell death caused by target chemicals was assayed with a lactate dehydrogenase (LDH) release assay kit from Beyotime Biotechnology (Nanjing, China) according to the description by manufacturer. A microplate reader was used to read the absorbance value of each prepared sample at 490 nm and the following fomula was used to calculate cell death ratio: Cell death (%) = (*A*_sample_ – *A*_control_/*A*_maximum_ – *A*_control_) × 100, in which *A* represented the absorbance value and *A*_maximum_ represented the absorbance value of the positive group.

### Assay of mitochondrial depolarization

The probe JC-1 obtained from Beyotime Biotechnology (Nanjing, China) was used to assay mitochondrial membrane potentials. In brief, after the cells treated with target chemicals were harvested, washed with PBS, incubated with JC-1 for 20 min at 37 °C, they were analyzed by flow cytometry (FACScan, Becton Dickinson, San Jose, CA, USA).

### Assay of superoxide onion, ROS and H_2_O_2_

The probe MitoSOX red from Invitrogen company (Eugene, OR, USA), the probe DCFH-DA and the H_2_O_2_ assay kit from Beyotime Biotechnology (Nanjing, China) were used respectively to measure mitochondrial superoxide, intracellular ROS and H_2_O_2_ as described by us previously [15]. The results were expressed as a ratio to the control cells.

### Transfection of small interfering RNA (siRNA)

siRNAs were introduced to cells by Lipofectamine 3000 (Invitrogen, USA). We obtained the siRNAs targeting PARP1 (5′-GAGACCCAAUAGGCUUAAUTT-3′), TAX1BP1 (5′-GCCUGAACAUUAUGUGGAATT-3′) and scrambled siRNA from GenePharma (Suzhou, China), and targeting ND1 siRNA (5′-CACTACAATCTTCCTAGGA-3′) from RiboBio (Guangzhou, China).

### Measurement of NADH oxidation by respiratory chain complex I

The activity of respiratory chain complex I in oxidizing NADH was assayed with a kit from Solarbio Science & Technology (Beijing, China) as described by the manufacture. Briefly, the collected cells were mixed with the agents supplied by manufacture, and then the absorbance of each sample at 340 nm was read at the time of 10 s and 2 min and recorded as *A*_1_ and *A*_2_ respectively. The complex I activity was calculated using the formula as follows,

$${{{{{{{\mathrm{Complex}}}}}}}}\;{{{{{{{\mathrm{I}}}}}}}}\;{{{{{{{\mathrm{activity}}}}}}}}\left( {{{{{{{{\mathrm{U}}}}}}}}/{{{{{{{\mathrm{mg}}}}}}}}\;{{{{{{{\mathrm{prot}}}}}}}}} \right) = \,	\left[ {{\Delta}{{{{{{A}}}}}} \times {{{{{{V_{{{\mathrm{rv}}}}}}}}}} \div \left( {{\upvarepsilon }} \times {{{{{{{\mathrm{d}}}}}}}} \right) \times 109} \right] \div \left( {{{{{{{V_{{{\mathrm{s}}}}}}}}}} \times {{{{{{C_{{{\mathrm{pr}}}}}}}}}}} \right) \div {{{{{{T}}}}}} \\ = \,	1608 \times {\Delta}{{{{{{A}}}}}} \div {{{{{{C_{{{\mathrm{pr}}}}}}}}}}{{{{{{{\mathrm{.}}}}}}}}$$

One unit of enzyme activity is defined as the amount of enzyme catalyzes the consumption of 1 nmol NADH per minute every milligram protein. ε: NADH molar extinction coefficient, 6.22 × 10^3^ L ·mol^−1^ ·cm^−1^; d: Light path of cuvette, 1 cm; *V*_rv_: Total reaction volume, 10^−3^ L; *V*_s_: Sample volume (mL), 0.05 mL; *C*_pr_: Sample protein concentration (mg/mL); *T*: Reaction time, 2 min.

### Assay of NAD^+^ level

NAD^+^ was assayed with a NAD^+^/NADH assay kit with WST-8 (Beyotime Biotechnology, China) as described by manufacture’s protocol. In brief, NAD^+^/NADH extraction buffer was added into collected cells or tumor tissue to make them lysis. For measurement of total NAD^+^/NADH level, 20 μL lysis solution was transferred into a 96 wells plate, incubated with 90 μL alcohol dehydrogenase for 10 min, followed by with 10 μL chromogenic solution for 30 min. The acquired absorbance value at wavelength 450 nm by a microplate reader was used to calculate the total level of NAD^+^/NADH following calibration to a standard concentration curve. For measurement of NADH level, the lysis solution was needed to be heated at 60 °C for 30 min followed by incubation with dehydrogenase and chromogenic solution. The amount of NAD^+^ was derived by subtracting NADH from total NAD^+^/NADH and expressed as a ratio to control group.

### Immunocytochemical staining

U87 cells seeded on a culture dish were treated with DPT 450 nmol/L for 24 h, incubated with 100 nmol/L Mito-tracker red for 30 min at 37 °C and then fixed in methanol. After being washed with PBS and blocked with 3% BSA, the cells were incubated respectively with Anti-TAX1BP1 (1:100), anti-AIF (1:100) or anti-ND1 (1:100) overnight at 4 °C, and then with Alexa Fluor 488-conjugated goat anti-rabbit IgG (1:1000) for 1 h followed by Hoechst 33258 (Beyotime Biotechnology, Nanjing, China) for 20 min. Eventually, confocal laser scanning microscopy (Olympus FV1000, Tokyo, Japan) was used to acquire cellular images.

### Differential centrifugation and Western blotting analyses

The differential centrifugation was used to isolate mitochondrial and nuclear fractions as reported previously by us [16]. The protein content of each fraction was assayed and normalized by using a BCA protein assay kit from Beyotime Biotechnology (Nanjing, China). Western blotting was also performed as reported previously [16]. Finally, proteins blots were visualized by using a Chemi-luminescence Developer (ChemiScope 5300, Clinx Scicence Instrument Company, Shanghai, China) and then quantified by ImageJ software.

### Coimmunoprecipitation

Coimmunoprecipitation was performed using immunoprecipitation kit with protein A + G magnetic beads (Beyotime Biotechnology, Nanjing, China) according to the instructions provided by manufacturer. Briefly, U87 cells were seeded onto a culture dish in a diameter of 10 cm. After treatment, they were harvested with lysis buffer by a scraper. The removed tumors tissues were homogenized with lysis buffer by a glass homogenizer. The lysed cells or tissues were centrifuged at 14,000 × *g* for 5 min at 4 °C to obtain the supernatant. Protein A + G magnetic beads bound with ND1 antibody or normal IgG was added in the ratio of 20 μL magnetic bead suspension per 500 μL normalized protein sample and incubated overnight at 4 °C. After magnetic separation and washed three times with lysis buffer, the protein A + G bead-antibody-antigen immune complexes were eluted and denatured using 100 μL SDS-PAGE sample loading buffer (1×) for each sample and heated for 5 min at 95 °C. After separation on magnetic rack for 10 s, the supernatant can be used for Western blotting.

### Human U87 tumor xenograft in mice

The athymic BALB/c nude mice (4 weeks; 20–22 g) from Beijing Vital River Laboratory were housed under a 12 h light and dark cycle with free access to water and food. The environment was pathogen-free and the research protocol was approved by the Ethics Committee of the First Hospital of Jilin University (Changchun, China). The U87 cells (1 × 10^6^) in 100 μL PBS were subcutaneously injected into the right flank of the mice. When the tumor volume reached about 150 mm^3^, the mice were intra-peritoneally injected once a day with 50 μL DPT at 10 mg/kg (*n* = 6) or vehicle (control group, *n* = 6) for consecutive 8 days. A slide caliper was used to measure the tumor length (A) and width (B) and then tumor volume was calculated according to the formula tumor volume = 0.5 × A × B^2^. When the therapy was terminated, cervical dislocation was used to euthanize the animals, and the removed tumor tissues were frozen immediately in liquid nitrogen.

### Assay of oxygen consumption rate (OCR)

The mitochondrial OCR was measured in real-time with Mito Stress Test Kit using the Seahorse XF analyzer (XFe24, Agilent) in vitro following manufacturer’s instructions. U87 cells were seeded in a specific 24-well plate at a density of 2.0 × 10^4^/well. After the target protein was silenced by siRNA and the cells were treated with 450 nmol/L DPT for 3 h, the medium was replaced with fresh DMEM, followed by addition of ATP synthase inhibitor oligomycin (10 μmol/L) to block proton movement and oxidative phosphorylation. Then, 2 mmol/L FCCP (carbonyl cyanide-4-(trifluoromethoxy) phenylhydrazone) was added to stimulate maximal OCR. Finally, rotenone (1 μmol/L) and antimycin (1 μmol/L) were added together to block complex I as well as compex III respectively in order to block mitochondrial respiration completely. The data were analyzed using Wave 2.6.1 software (Agilent Technologies Inc, CA, USA).

### Real-time PCR assay (RT-qPCR)

Total RNA in U87 cells was extracted by TRIzol reagent (Invitrogen, CA, USA). cDNA was synthesized using the First-Strand cDNA Synthesis Super Mix (TransGen Biotech, Beijing, China) and quantitative real-time PCR was performed using PerfectStart Green qPCR SuperMix (TransGen Biotech, Beijing, China) in a CFX96 Real time PCR machine (BIO-RAD, Kyoto, Japan). The results were expressed as a ratio to control group. The primer sequences were:

TAX1BP1: Forward: 5′-AAGAAACAGCACAACTTCGAGA-3′,

Reverse: 5′-TGGATGTAGCATCACTGAACCT-3′,

β-actin: Forward: 5′-CAGGTCATCACCATTGGCAATGAGC -3′,

Reverse: 5′-CGGATGTCCACGTCACACTTCATGA-3′.

### Statistical analysis

The obtained data were expressed as mean ± SD. One-way ANOVA was used to make statistical comparisons. *P* values of <0.05 were considered to be statistically significant.

## Results

### TAX1BP1 contributed to DPT-induced nuclear translocation of AIF

**Fig. 1:**
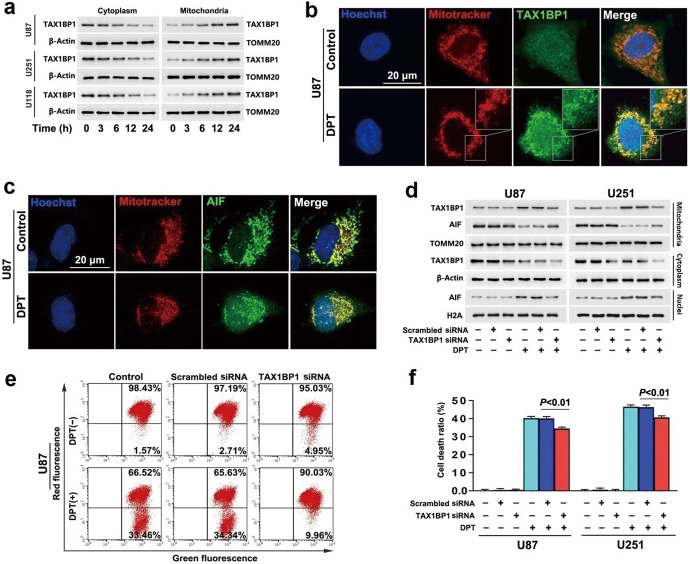
TAX1BP1 contributed to DPT-induced nuclear translocation of AIF. **a** Western blotting revealed that treatment with DPT at 450 nmol/L induced a reduction in cytoplasmic TAX1BP1, but improved mitochondrial TAX1BP1 in a time-dependent manner. **b** Representative images of confocal microscopy combined with immunochemical staining showed that TAX1BP1 accumulated apparently in mitochondria of the U87 cells treated with 450 nmol/L DPT for 24 h, when compared with that in control cells. **c** Representative images of confocal microscopy combined with immunochemical staining showed that AIF decreased in mitochondria, but accumulated apparently in nucleus of the U87 cell treated with 450 nmol/L DPT for 24 h. **d** Western blotting revealed that the increased TAX1BP1 and the decreased AIF in mitochondrial fractions caused by 450 nmol/L DTP at 24 h were both obviously inhibited when TAX1BP1 was knocked down with siRNA. Concomitantly, the upregulated AIF in nuclear fractions was prevented as well. **e** Flow cytometry analysis with JC-1 staining showed that the depleted mitochondrial membrane potentials caused by 450 nmol/L DPT at 24 h was apparently inhibited when TAX1BP1 was knocked down with siRNA. **f** LDH release assay showed that knockdown of TAX1BP1 with siRNA significantly prevented the glioma cell death induced by 450 nmol/L DPT at 24 h. The values are expressed as mean ± SD (*n* = 5 per group).

To investigate DPT-induced changes of TAX1BP1 in glioma cells, the U87, U251 and U118 cells were treated with 450 nmol/L DPT as reported previously by us [14]. After the cells were treated for 6 h, 12 h and 24 h, we extracted mitochondrial and cytoplasmic fractions by using differential centrifugation and used Western blotting to examine the protein level of TAX1BP1 in each fraction. Compared with that in control cells, cytoplasmic TAX1BP1 was decreased whereas mitochondrial TAX1BP1 was increased by DPT time-dependently (Fig. 1a). Consistently, confocal microscopy also showed that TAX1BP1 accumulated more obviously on the mitochondria of the cells treated with 450 nmol/L DPT for 24 h than that of control group (Fig. 1b). To test the effect of DPT on TAX1BP1 transcription, we extracted total RNA from the U87 cells incubated with 450 nmol/L DPT and then performed RT-qPCR. In comparison with that in control group, the mRNA level of TAX1BP1 was increased by DPT at 12 h, and further increased when treatment time was extended to 24 h (Supplementary Fig. S1a). Therefore, these results suggested that DPT not only increased the transcription of TAX1BP1, but also boosted TAX1BP1 translocation from cytoplasm to mitochondria.

Then, confocal microscopy showed that nuclear AIF was obviously higher in the U87 cells incubated with 450 nmol/L DPT for 24 h than that in control cells (Fig. 1c). Furthermore, kinetic analysis of DPT-induced changes in AIF distribution by using Western blotting revealed that DPT treatment resulted in time-dependent translocation of AIF from mitochondria to nuclei (Fig. 1d). JC-1 is a probe emitting red fluorescence when mitochondrial membrane potential is at higher level, but green fluorescence when mitochondrial membrane potential decreases [3]. Thus, we used JC-1 to detect DPT-induced changes in mitochondrial membrane potential, which controls mitochondrial release of AIF [3]. As shown by flow cytometry, the red fluorescence decreased obviously in the cells treated with DPT at 450 nmol/L for 24 h in comparison with that in control ones (Fig. 1e). These indicated that DPT induced nuclear translocation of AIF by causing mitochondrial depolarization. To address the relationship between TAX1BP1 accumulation in mitochondrial and nuclear translocation of AIF, siRNA was introduced to decrease DPT-induced TAX1BP1 improvement in mitochondria. It was found that knockdown of TAX1BP1 with siRNA not only decreased the level of TAX1BP1 mRNA (Supplementary Fig. S1b), but also inhibited DPT-induced increase of TAX1BP1 in mitochondrial fraction (Fig. 1d). Moreover, DPT-induced mitochondrial depolarization, nuclear translocation of AIF, and glioma cell death were all attenuated when TAX1BP1 was knocked down (Fig. 1d–f). Notably, we found that the increased mitochondrial oxygen consumption rate and ATP generation caused by treatment with DPT at 450 nmol/L for 3 h was also significantly inhibited by knocking down of TAX1BP1 (Supplementary Fig. S1c). These results suggested TAX1BP1 exacerbated DPT-induced nuclear translocation of AIF in glioma cells, which might be associated with regulating the function of mitochondrial respiratory chain.

### TAX1BP1 boosted DPT-induced accumulation of mitochondrial superoxide

**Fig. 2:**
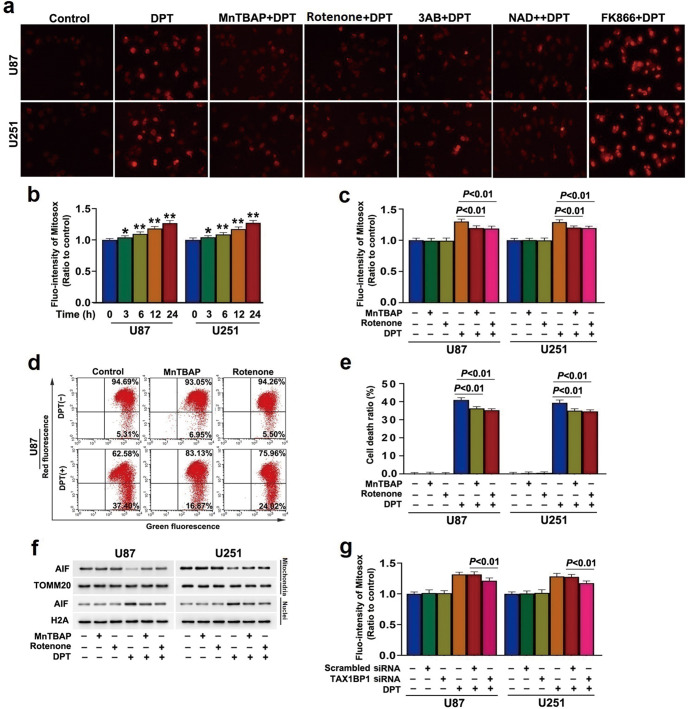
TAX1BP1 boosted DPT-induced mitochondrial generation of superoxide. **a** Representative images of glioma cells incubated with mitochondrial superoxide probe MitoSOX red under fluorescence microscope (20×). Treatment with 450 nmol/L DPT for 24 h resulted in significant improvement in the red fluorescence, which was mitigated in the cells pretreated for 1 h with 40 μmol/L MnTBAP, 2 μmol/L rotenone, 500 μmol/L 3AB and 2 mmol/L exogenous NAD^+^ respectively, but aggravated by pretreatment with 200 μmol/L FK866 for 1 h. **b** Statistical analysis of the red fluorescence intensity exhibited by MitoSOX red proved that 450 nmol/L DPT triggered mitochondrial accumulation of superoxide in a time-dependent manner. **c** Statistical analysis revealed that pretreatment with 40 μmol/L MnTBAP or 2 μmol/L rotenone for 1 h suppressed the increase of mitochondrial superoxide caused by 450 nmol/L DPT at 24 h. **d** Flow cytometry analysis with JC-1 staining showed the depletion of mitochondrial membrane potential caused by 450 nmol/L DPT at 24 h was obviously inhibited in the presence of 40 μmol/L MnTBAP or 2 μmol/L rotenone. **e** LDH release assay showed that the glioma cell death caused by 450 nmol/L DPT at 24 h was obviously inhibited by 40 μmol/L MnTBAP or 2 μmol/L rotenone. **f** Western blotting analysis revealed that DPT-induced nuclear translocation of AIF was inhibited when the cells were pretreated with 40 μmol/L MnTBAP or 2 μmol/L rotenone. **g** Statistical analysis of the intensity of the red fluorescence detected by MitoSOX red revealed that DPT-induced upregulation of mitochondrial superoxide was suppressed when TAX1BP1 was knocked down with siRNA. *P < 0.05 versus control group; **P < 0.01 versus control groups. The values are expressed as mean ± SD (*n* = 5 per group).

Considering that accumulated mitochondrial superoxide could cause AIF translocation from mitochondria to nuclei by decreasing mitochondrial membrane potentials [7], we examined whether DPT treatment resulted in accumulation of mitochondrial superoxide by using MitoSOX red probe and found the red fluorescence in the cells treated with 450 nmol/L DPT for 24 h were much brighter than that in control ones (Fig. 2a). Statistical analysis showed as well that DPT induced time-dependent accumulation of mitochondrial superoxide in glioma cells (Fig. 2b). To address the role of mitochondrial superoxide in DPT-induced mitochondrial damage and glioma cell death, the cells were treated 1 h with 40 μmol/L MnTBAP, a mimic of mitochondrial superoxide dismutase and often used to mitigate mitochondrial superoxide, and then incubated with 450 nmol/L DPT for 24 h. It was found that MnTBAP not only mitigated DPT-induced mitochondrial superoxide accumulation, but also reversed mitochondrial depolarization, nuclear translocation of AIF and cell death (Fig. 2c–f). These indicated that DPT induced mitochondrial damage and glioma cell death by triggering accumulation of mitochondrial superoxide. Furthermore, the accumulated mitochondrial superoxide caused by DPT was significantly suppressed in cells transfected with TAX1BP1 siRNA (Fig. 2g). These results showed that TAX1BP1 contributed to DPT-induced mitochondrial damage by promoting mitochondrial accumulation of superoxide.

### TAX1BP1 contributed to DPT-induced activation of complex I

**Fig. 3:**
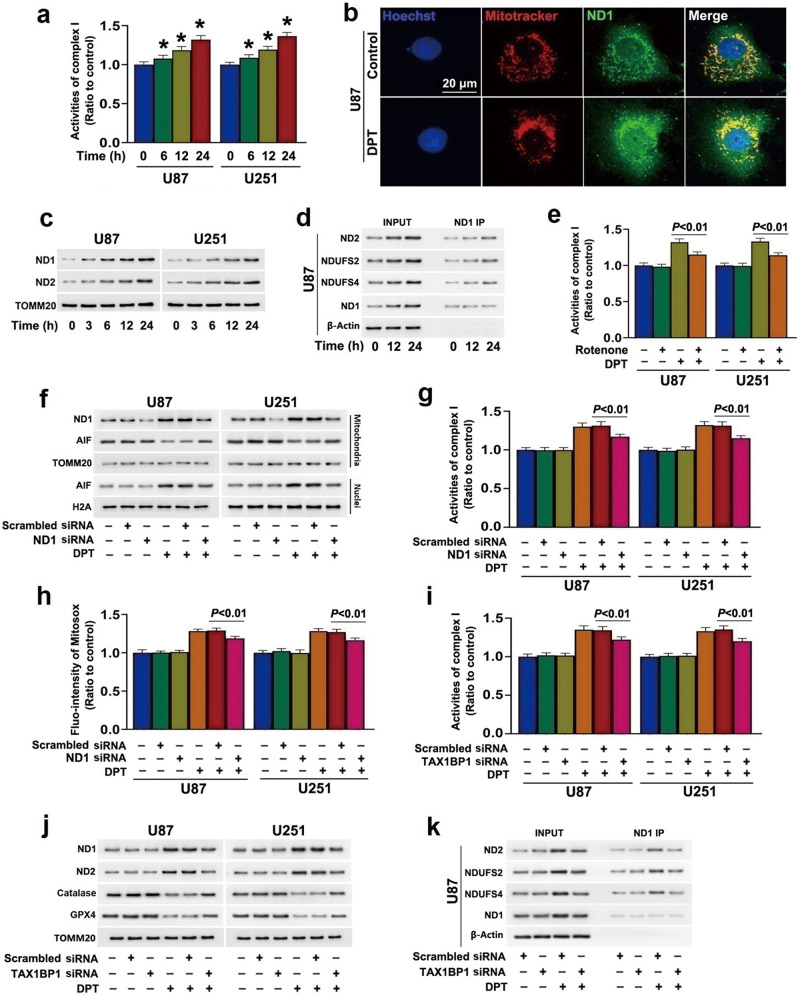
TAX1BP1 promoted DPT-induced activation of complex I. **a** Complex I activity assay showed that 450 nmol/L DPT enhanced complex I activity in oxidation of NADH in a time-dependent manner. **b** Representative images of confocal microscopy combined with immunochemical staining showed ND1 level was increased obviously in the mitochondria of the cells incubated with 450 nmol/L DPT for 24 h. **c** Western blotting analysis revealed 450 nmol/L DPT upregulated both ND1 and ND2 in a time-dependent manner. **d** Co-immunoprecipitation analysis showed that the protein levels of ND2, NDUFS2 and NDUFS4 co-immunoprecipitated with ND1 were increased by 450 nmol/L DPT at 12 h, which became more apparent when incubation time of DPT was increased to 24 h. **e** Complex I activity assay showed that the increased complex I activity in oxidation of NADH caused by 450 nmol/L DPT at 24 h was inhibited by pretreatment with 2 μmol/L rotenone for 1 h. **f** Western blotting revealed that knockdown of ND1 with siRNA not only apparently inhibited ND1 upregulation in mitochondria, but also alleviated nuclear translocation of AIF induced by 450 nmol/L DPT at 24 h. **g** Complex I activity assay showed that the increased complex I activity in oxidation of NADH caused by 450 nmol/L DPT at 24 h was inhibited in the cells transfected with ND1 siRNA. **h** Statistical analysis of the intensity of the red fluorescence detected by MitoSOX red revealed that the increased mitochondrial superoxide induced by 450 nmol/L DPT at 24 h was suppressed when ND1 was knocked down with siRNA. **i** Complex I activity assay showed that knockdown of ND1 with siRNA significantly prevented the increase of complex I activity in oxidation of NADH caused by 450 nmol/L DPT at 24 h. **j** Western blotting revealed knockdown of TAX1BP1 with siRNA prevented the upregulation of ND1 and ND2 and downregulation of GPX4 and catalase in mitochondrial fractions induced by 450 nmol/L DPT at 24 h. **k** Co-immunoprecipitation analysis showed that the enhanced interactions between ND1, ND2, NDUFS2 and NDUFS4 caused by 450 nmol/L DPT at 24 h were all inhibited when TAX1BP1 was knocked down with siRNA. *P < 0.01 versus control group. The values are expressed as mean ± SD (*n* = 5 per group).

Mitochondrial respiratory chain complex I is a main site of superoxide generation, where the electrons formed due to NADH oxidation to NAD^+^ could escape from complex I and combine directly with molecular oxygen to form superoxide [6]. Moreover, DPT treatment could enhance mitochondrial respiration, we thus assayed the effect of DPT on complex I activity to oxidize NADH. It was found the activity of complex I was upregulated when the cells were treated with 450 nmol/L DPT for 6 h, and was further increased when treatment time was extended to 12 and 24 h (Fig. 3a). Then, the protein levels of complex I core subunits ND1 and ND2 were analyzed by Western blotting. As shown in Fig. 3c, both ND1 and ND2 levels were increased time-dependently in the cells treated with 450 nmol/L DPT. Confocal microscopy combined with immunochemical staining of ND1 also showed that the green fluorescence of ND1 in the U87 cells treated with DPT for 24 h was much brighter than that in control group (Fig. 3b). Then, immunoprecipitation was used to test whether DPT could enhance the interaction between ND1 and other subunits of complex I. As shown in Fig. 3d, the levels of ND2, NDUFS2 and NDUFS4 co-immunoprecipitated with ND1 were all increased with the extension of DPT incubation time. These results indicated that DPT increased the assemblies of these subunits into complex I.

To test the role of activated complex I in DPT-induced cell death, we treated the cells with complex I inhibitor rotenone at 2 μmol/L for 1 h before incubation with 450 nmol/L DPT. As shown in Fig. 3e, rotenone prevented the enhanced complex I activity in oxidation of NADH caused by DPT. In the meantime, DPT-induced mitochondrial superoxide accumulation, mitochondrial depolarization, AIF nuclear translocation and cell death were all mitigated by rotenone (Fig. 2c–f). To further verify the role of activated complex I in regulation of mitochondria damage, we knocked down ND1 level with siRNA. It was found that ND1 knockdown not only prevented DPT-induced increases in complex I activity, but also inhibited superoxide accumulation and AIF nuclear translocation (Fig. 3f–h). Notably, the increased complex I activity in oxidation of NADH and expressional upregulation of ND1 and ND2 caused by DPT were all inhibited by knockdown of TAX1BP1 with siRNA (Fig. 3i, j). Immunoprecipitation of ND1 further showed TAX1BP1 knockdown also mitigated the enhanced interaction between ND1 and other complex I subunits resulting from DPT treatment (Fig. 3k). These results indicated that TAX1BP1 contributed to the activation and assembly of complex I caused by DPT.

In addition, we investigated the effect of DPT on mitochondrial catalase and GPX4, because they both could inhibit mitochondria ROS [17]. As shown in Fig. 3j, both catalase and GPX4 were downregulated in cells treated with 450 nmol/L DPT for 24 h. In contrast, the decreased levels of catalase and GPX4 induced by DPT were both prevented when TAX1BP1 was knocked down (Fig. 3j). These indicated TAX1BP1 also contributed to DPT-induced superoxide accumulation by downregulation of catalase and GPX4.

### PARP1 regulated DPT-induced mitochondrial translocation of TAX1BP1 in glioma cells

**Fig. 4:**
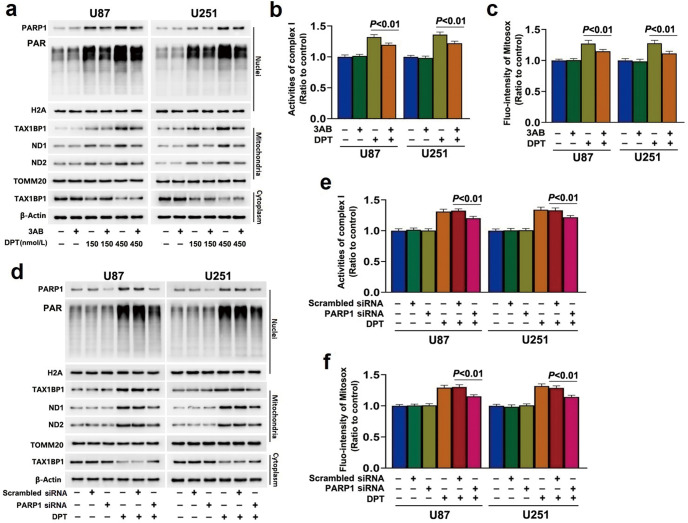
PARP1 regulated DPT-induced mitochondrial translocation of TAX1BP1 in glioma cells. **a** Western blotting showed that pretreatment with 500 μmol/L 3AB for 1 h obviously prevented the upregulation of nuclear PARP1, PAR, mitochondrial TAX1BP1, ND1, ND2 and downregulation of cytoplasmic TAX1BP1 triggered by 450 nmol/L DPT at 24 h. **b** Complex I activity assay showed that the increased complex I activity in oxidation of NADH caused by 450 nmol/L DPT at 24 h was inhibited in the cells pretreated with 500 μmol/L 3AB for 1 h. **c** Statistical analysis of the intensity of the red fluorescence detected by MitoSOX red proved that pretreatment with 500 μmol/L 3AB for 1 h obviously prevented the increase of mitochondrial superoxide induced by 450 nmol/L DPT at 24 h. **d** Western blotting showed that the upregulated PARP1 and PAR in nuclear fractions, TAX1BP1, ND1 and ND2 in mitochondrial fractions and the downregulated TAX1BP1 in cytoplasmic fractions induced by 450 nmol/L DPT at 24 h were all inhibited when PARP1 was knocked down with siRNA. **e** Complex I activity assay showed that the increased complex I activity caused by 450 nmol/L DPT at 24 h was inhibited by knocking down PARP1 with siRNA. **f** Statistical analysis of the intensity of the red fluorescence detected by MitoSOX red revealed that knockdown of PARP1 with siRNA significantly prevented the increase of mitochondrial superoxide caused by 450 nmol/L DPT at 24 h. The values are expressed as mean ± SD (*n* = 5 per group).

Because over-activated PARP1 contributes to DPT-induced glioma cell death [14], we tested whether PARP1 could regulate TAX1BP1 distribution to mitochondria. Western blotting showed pretreatment with PARP-1 inhibitor 3AB at 500 μmol/L for 1 h obviously inhibited DPT-induced TAX1BP1 increase in mitochondria and reduction in cytoplasm, as well as restrained the upregulation of PARP1 and PAR (Fig. 4a). Meanwhile, pretreatment with 3AB obviously inhibited DPT-induced increases of mitochondrial superoxide and complex I activity and expressional upregulation of ND1 and ND2 (Fig. 4a–c). To further clarify the role of PARP-1 in DPT-induced mitochondrial translocation of TAX1BP1 and superoxide accumulation, siRNA was introduced to knock down PARP1. It was found that knockdown of PARP1 with siRNA obviously prevented DPT-triggered increase of TAX1BP1 in mitochondria, accumulation of mitochondrial superoxide, improvement of complex I activity and upregulation of ND1 and ND2 (Fig. 4d–f). These results suggested PARP1 regulated DPT-induced TAX1BP1 mitochondrial translocation.

### NAD^+^ depletion promoted TAX1BP1 accumulation on mitochondria

**Fig. 5:**
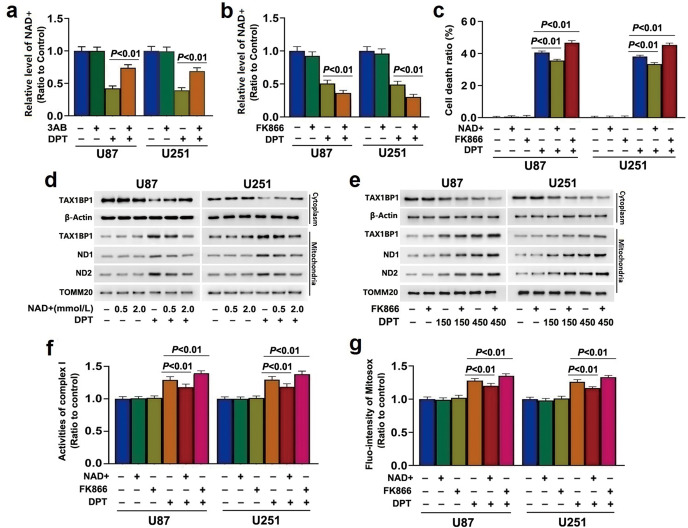
NAD^+^ depletion promoted TAX1BP1 accumulation on mitochondria. **a** and **b** NAD^+^ assay showed the NAD^+^ depletion caused by 450 nmol/L DPT at 24 h was significantly prevented by prior incubation with 500 μmol/L 3AB for 1 h, but aggregated by pretreatment with 100 μmol/L FK866 for 1 h. **c** LDH release assay demonstrated that the glioma cell death induced by 450 nmol/L DPT at 24 h was significantly prevented by pretreatment with exogenous NAD^+^ at 2 mmol/L for 1 h, but aggregated by incubation with 200 μmol/L FK866 for 1 h. **d** Western blotting showed that incubation with exogenous 2 mmol/L NAD^+^ for 1 h obviously suppressed the upregulation of TAX1BP1, ND1 and ND2 in mitochondrial fractions and the downregulation of TAX1BP1 in cytoplasmic fractions caused by 450 nmol/L DPT at 24 h. **e** Western blotting proved that DPT-induced upregulation of TAX1BP1, ND1 and ND2 in mitochondrial fractions and downregulation of TAX1BP1 in cytoplasmic fractions were all exacerbated by incubation with 200 μmol/L FK866 for 1 h. **f** Complex I activity assay showed that the increased complex I activity in oxidation of NADH caused by 450 nmol/L DPT at 24 h was inhibited in the cells incubated with exogenous 2 mmol/L NAD^+^ for 1 h, but exacerbated by treatment with 200 μmol/L FK866 for 1 h. **g** Statistical analysis of the intensity of the red fluorescence detected by MitoSOX red revealed that the increase of mitochondrial superoxide caused by 450 nmol/L DPT at 24 h was inhibited by NAD^+^, but exacerbated by FK866. The values are expressed as mean ± SD (*n* = 5 per group).

To clarify why over-activated PARP1 could promote TAX1BP1 distribution to mitochondria, we tested the role of NAD^+^ in DPT-induced glioma cell death considering that it is a substrate of activated PARP1 [18]. As shown in Fig. 5a, NAD^+^ level was decreased in the cells treated with 450 nmol/L DPT for 24 h, which was prevented by pretreatment of 3AB at 500 μmol/L for 1 h. This indicated that PARP1 activation led to NAD^+^ depletion in DPT-treated cells. To clarify whether NAD^+^ depletion promoted DPT-induced cell death, cells were pretreated for 1 h with 200 μmol/L FK866, a potent inhibitor of nicotinamide phosphoribosyltransferase which is the rate-limiting enzyme for NAD^+^ regeneration, and then incubated with DPT at 450 nmol/L for 24 h. It was found pretreatment of FK866 aggravated DPT-induced NAD^+^ depletion (Fig. 5b). LDH release assay revealed FK866 at 200 μmol/L did not show toxicity in glioma cells, but aggravated DPT-induced cell death (Fig. 5c). In contrast, the cell death caused by DPT was inhibited when cells were pretreated with exogenous NAD^+^ at 2 mmol/L for 1 h (Fig. 5c). These results suggested that NAD^+^ depletion contributed to DPT-induced glioma cell death. Notably, supplement of exogenous NAD^+^ suppressed, but pretreatment with FK866 aggravated DPT-induced mitochondrial TAX1BP1 increase and cytoplasmic TAX1BP1 reduction (Fig. 5d, e). In the meantime, DPT-triggered upregulation of ND1 and ND2, accumulation of mitochondrial superoxide and increase of complex I activity were all inhibited by supplemental NAD^+^, but exacerbated by FK866 (Fig. 5d–g). Thus, we demonstrated that NAD^+^ depletion caused by over-activated PARP1 contributed to DPT-induced TAX1BP1 translocation to mitochondria.

### Mitochondrial superoxide exacerbated PARP1 activation by promoting DNA damage

**Fig. 6:**
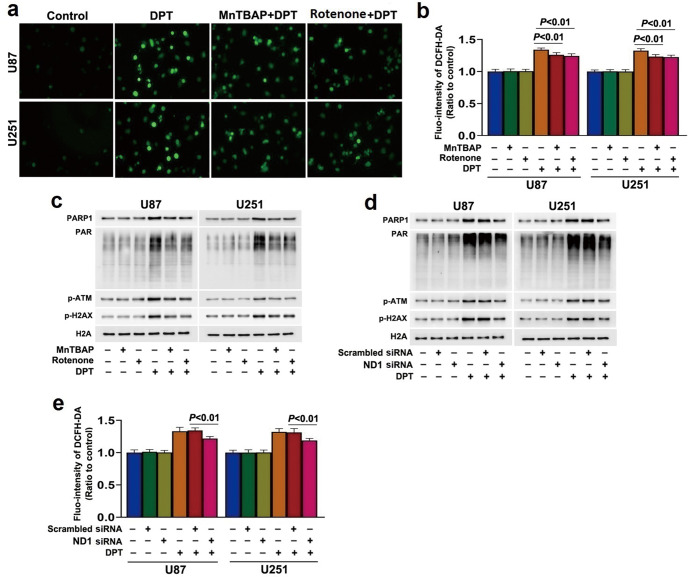
Mitochondrial superoxide exacerbated PARP1 activation by promoting DNA damage. **a** Representative images of glioma cells incubated with DCFH-DA probe under fluorescence microscope (20×). When compared with control cells, treatment with DPT at 450 nmol/L for 24 h resulted in significant improvement in the green fluorescence, which was mitigated by pretreatment with 40 μmol/L MnTBAP or 2 μmol/L rotenone for 1 h. **b** Statistical analysis of the intensity of the green fluorescence detected by DCFH-DA probe revealed that DPT-induced ROS accumulation was inhibited in the presence of MnTBAP or rotenone. **c** Western blotting showed that the upregulated nuclear PARP1, PAR, p-ATM and p-H2AX caused by 450 nmol/L DPT at 24 h were all inhibited by pretreatment with 40 μmol/L MnTBAP or 2 μmol/L rotenone for 1 h. **d** Western blotting showed that the upregulated PARP1, PAR, p-ATM and p-H2AX in nuclear fractions caused by 450 nmol/L DPT at 24 h were all inhibited when ND1 was knocked down with siRNA. **e** Statistical analysis of the intensity of the green fluorescence detected by DCFH-DA probe revealed that the increased ROS caused by 450 nmol/L DPT at 24 h was significantly inhibited by knocking down of ND1 with siRNA. The values are expressed as mean ± SD (*n* = 5 per group).

Excessive mitochondrial superoxide could lead to intracellular accumulation of ROS, we thus examined the effect of DPT on intracellular ROS levels using ROS probe DCFH-DA. It was found that the green fluorescence exhibited by DCFH-DA in the cells treated with 450 nmol/L for 24 h was much brighter than that in control cells, which was further verified by statistical analysis (Fig. 6a, b). Pretreatment with 40 μmol/L MnTBAP significantly reduced the increases of ROS caused by DPT (Fig. 6a, b). This indicated that the increased mitochondrial superoxide caused by DPT led to intracellular accumulation of ROS.

Considering that ROS could activate PARP1 by causing DNA-double strand breaks (DSBs) [19, 20], we investigated the role of ROS in DPT-induced DNA strand break and PARP1 activation. Western blotting showed treatment with 450 nmol/L DPT for 24 h not only apparently increased the level of γ-H2AX (phosphos-H2AX at Ser139) which is a prominent biomarker of DNA DSBs, but also upregulated the protein level of p-ATM (phospho-ataxia telangiectasia mutated at Ser1981) which accounts for γ-H2AX formation (Fig. 6c). At the same time, the levels of PARP1 and PAR were both improved in DPT-treated cells (Fig. 6c). Importantly, the accumulation of ROS and upregulation of γ-H2AX, p-ATM, PARP1 and PAR induced by DPT were all abrogated in the cells pretreated with 40 μmol/L MnTBAP for 1 h (Fig. 6b, c). Moreover, inhibition of complex I activity with 2 μmol/L rotenone or by knocking down ND1 with siRNA significantly alleviated DPT-induced ROS accumulation and upregulation of γ-H2AX, p-ATM, PARP1 and PAR (Fig. 6b–e). These results showed that the superoxide generated by complex I with increased activity exacerbated PARP1 activation via exacerbation of DNA DSBs.

### DPT triggered NAD^+^ depletion and mitochondrial translocation of TAX1BP1 in vivo

**Fig. 7:**
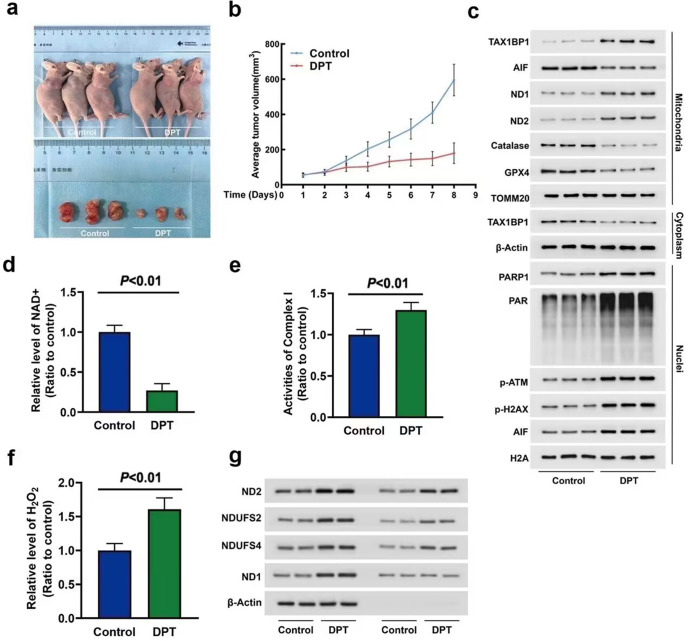
DPT triggered NAD^+^ depletion and mitochondrial translocation of TAX1BP1 in vivo. **a** Representative images of nude mice with xenografted gliomas showed that tumor growth was significantly inhibited when the mice were treated with DPT at a dose of 10 mg/kg for 8 consecutive days. **b** Statistical analysis of the tumor volumes confirmed that the growth of the tumor in vivo was significantly inhibited by DPT. **c** Western blotting analysis revealed that DPT promoted mitochondrial translocation of TAX1BP1 and nuclear translocation of AIF. It also induced marked upregulation of ND1 and ND2 and downregulation of mitochondrial GPX4 and catalase in mitochondrial fractions, and obvious upregulation of PARP1, PAR, p-ATM and p-H2AX in nuclear fractions. **d** NAD^+^ assay showed that NAD^+^ was decreased significantly in DPT-treated group when compared with that in control group. **e** Complex I activity assay showed that DPT treatment resulted in upregulation of complex I activity in oxidation of NADH in vivo. **f** H_2_O_2_ assay proved the level of H_2_O_2_ was increased obviously by DPT in vivo. **g** Immunoprecipitation with an antibody against ND1 revealed that the co-immunoprecipitated ND2, NDUFS2 and NDUFS4 with ND1 were all significantly increased in the DPT-treated group. The values are expressed as mean ± SD (*n* = 6 per group).

To verify the therapeutical effect of DPT on glioma in vivo, we established xenografted glioma model by injecting subcutaneously U87 cells into the right flank of mice, and then the mice were treated once a day with DPT at 10 mg/kg for consecutive 8 days. It was found that the tumor growth began to be inhibited after the mice have been treated with DPT for 3 days and treatment with DPT for 8 days made tumor volume being significantly less than that in control group (Fig. 7a, b). Western blotting showed that DPT treatment increased the levels of mitochondrial TAX1BP1 and nuclear AIF, but decreased the levels of cytoplasmic TAX1BP1 and mitochondrial AIF (Fig. 7c). Compared with control group, the NAD^+^ levels in the tumors removed from DPT-treated mice were obviously suppressed (Fig. 7d). These results suggested DPT inhibited the growth of glioma and caused NAD^+^ depletion, TAX1BP1 distribution to mitochondria and AIF distribution to nuclei in vivo.

Then, we investigated whether DPT promoted the activity and assembly of complex I and induced ROS accumulation in vivo. The complex I activity and the protein levels of ND1 and ND2 were all increased by DPT (Fig. 7c, e). Immunoprecipitation of ND1 with its antibody further confirmed that treatment of DPT enhanced the interactions between ND1 and other subunits of complex I including ND2, NDUFS2 and NDUFS4 (Fig. 7g). Treatment of DPT also increased H_2_O_2_ levels and caused expressional downregulation of GPX4 and catalase (Fig. 7c, f). These data indicated that DPT induced ROS accumulation by promoting complex I activity and assembly and downregulating GPX4 and catalase. Moreover, the levels of p-ATM, p-H2AX, PARP1 and PAR were all increased in DPT-treated gliomas (Fig. 7c). These data indicated DPT triggered DNA DSBs and PARP1 activation in vivo.

## Discussion

**Fig. 8:**
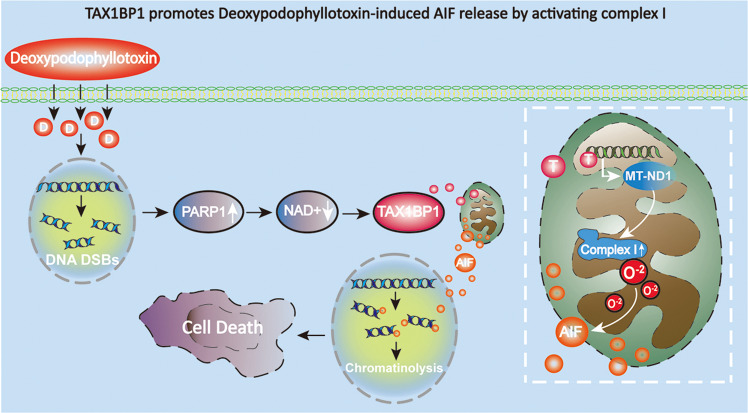
Schematic diagram of the role of TAX1BP1 in DPT-induced parthanatos. DPT induces PARP1 over-activation via causing DNA double strand breaks (DSBs). The activated PARP1 promotes TAX1BP1 translocation from cytoplasm to mitochondria by depletion of its substrate NAD^+^ . Then, TAX1BP1 enhances the activation of respiratory chain complex I not only by increasing the protein levels of ND1 and ND2, but also via promoting their assemblies into complex I. The activated respiratory chain complex I generates more superoxide, resulting AIF translocation from mitochondria to nuclei. Within nuclei, AIF serves as a nuclease to cause chromatinolysis to complete the final stage of parthanatos.

In summary, we found in the present study that DPT induced PARP1 over-activation and TAX1BP1 distribution to mitochondria in the glioma cells in vitro and in vivo. In vitro studies revealed PARP1 activation resulted in TAX1BP1 distribution to mitochondria by depleting nicotinamide adenine dinucleotide (NAD^+^). Knockdown of TAX1BP1 with siRNA not only inhibited TAX1BP1 accumulation on mitochondria, but also alleviated nuclear translocation of AIF and glioma cell death. Thus, TAX1BP1 is a downstream signal of activated PARP1 and triggers AIF translocation from mitochondria to nuclei. Mechanistically, TAX1BP1 enhanced the activity of respiratory chain complex I not only by upregulating the expression of ND1, ND2, NDUFS2 and NDUFS4, but also promoting their assemblies into complex I. Then, the activated respiratory complex I generated more superoxide to cause mitochondrial depolarization and nuclear translocation of AIF. Furthermore, the increased mitochondrial superoxide reversely reinforced PARP1 activation by inducing ROS-dependent DNA double strand breaks. Taken together, these data indicated that TAX1BP1 acts as a downstream signal of activated PARP1 to trigger nuclear translocation of AIF by activation of mitochondrial respiratory chain complex I (Fig. 8).

During the process of parthanatos, AIF translocation from mitochondria to nuclei is a key biochemical event leading to cell death. It is well accepted that PAR polymers account for the nuclear translocation of AIF triggered by activated PARP1 [4, 5]. However, further studies revealed the NAD^+^ depletion resulting from PARP1-dependent synthesis of PAR polymers might also be a factor initiating nuclear translocation of AIF. It was reported supplement of exogenous NAD^+^ not only protected mitochondria against the damage caused by calcium overload [21], but also inhibited AIF release from mitochondria and distribution to nuclei [22]. These findings verify that NAD^+^ deletion could trigger nuclear translocation of AIF. In this study, we found PARP1 activation not only promoted nuclear translocation of AIF, but also resulted in NAD^+^ depletion. Supplement of exogenous NAD^+^ alleviated, but exacerbation of NAD^+^ depletion with FK866 aggravated AIF translocation from mitochondria to nuclei. Thus, NAD^+^ depletion contributed to PARP1-triggered nuclear translocation of AIF in glioma cells.

Although Bax, Bim and Noxa could induce nuclear translocation of AIF upon accumulation on mitochondria [23, 24], it remains elusive of the signals downstream of NAD^+^ depletion to induce AIF translocation from mitochondria to nuclei. As a protein that could insert its C-terminal domain into mitochondrial outer membrane, BNIP3 accumulation on mitochondria was found to be induced by NAD^+^ depletion. It was reported that hypoxia-induced NAD^+^ depletion triggered nuclear translocation of AIF in neurons by promotion of BNIP3 distribution to mitochondria [25]. TAX1BP1 is also a protein targeting mitochondria, because it could directly bind with MAVS, an adaptor protein located on mitochondrial outer membrane [26]. Thus, TAX1BP1 could potentially regulate nuclear translocation of AIF, despite it often serves as autophagy receptor [27]. In this study, we found DPT did not alter the expression of TAX1BP1, but promoted its distribution to mitochondria. Knockdown of TAX1BP1 with siRNA not only attenuated the increase of TAX1BP1 in mitochondria, but also alleviated nuclear translocation of AIF. Supplement of exogenous NAD^+^ inhibited, but exacerbation of NAD^+^ depletion with FK866 aggravated TAX1BP1 accumulation on mitochondria. These verify that TAX1BP1 is a downstream signal of NAD^+^ depletion and causes nuclear translocation of AIF in the glioma cells undergoing parthanatos.

Mitochondrial superoxide plays a crucial role in promoting nuclear translocation of AIF in glioma cells. Consistent with previous reports showing that mitochondrial superoxide contributed to shikonin- and silibinin-induced nuclear translocation of AIF in glioma cells [7, 16], the data in this study proved mitigation of mitochondrial superoxide with MnTBAP suppressed DPT-induced AIF translocation into nuclei. Mitochondria superoxide formation is primarily due to molecular oxygen binding with the electrons escaped from respiratory chain complex I [28]. The electrons formed during the course of NADH oxidization to NAD^+^ could escape from complex I at the flavin binding site and the ubiquinone-binding site [29]. As an inhibitor of respiratory chain complex I, rotenone improves mitochondrial superoxide by enhancing electron leakage at the flavin binding site due to blocking ubiquinone-binding site [29]. However, it could also attenuate mitochondrial superoxide generated by complex I via two pathways. One is inhibiting NADH oxidation to NAD^+^ to suppress electron entry into complex I and the other is to alleviate electron leakage at ubiquinone-binding site by blocking reverse electron flow from CoQH_2_ driven by proton motive force or mitochondrial hyper-polarization [30]. In this study, rotenone was found to inhibit DPT-induced mitochondrial depolarization and upregulation in complex I activity to oxidize NADH, as well as attenuate superoxide accumulation. Notably, knockdown of TAX1BP1 with siRNA obviously prevented NADH oxidation by complex I and superoxide accumulation. Thus, TAX1BP1 contributed to DPT-induced mitochondrial superoxide generation by activation of respiratory chain complex I.

The respiratory chain complex I is composed of 45 subunits with a molecular weight close to 1000 kDa, seven of which such as ND1 and ND2 are core subunits encoded by mitochondrial DNA, and the others such as NFUFS2 and NDUFS4 are auxiliary subunits encoded by nuclear DNA [6]. As a core subunit of complex I, ND1 mutation could disrupt the assembly of complex I at early stage, with complex I-linked respiration reduction to 2.0% of control levels [31]. However, stable transduction of yeast complex I subunit Ndi1 was found to reinforce complex I activity in breast cancer cells [32]. In addition, complex I activity could also be increased when its auxiliary subunit is upregulated, given that S100 calcium-binding protein A4 was reported to enhance complex I activity in lung cancer cells by upregulation of NDUFS2 [33]. Further studies showed that complex I activation produced dual effects on cancer cells’ destiny. It promoted colorectal cancer cell growth [34], but inhibited breast cancer cells’ growth and metastasis [35]. In this study, we found mitochondrial translocation of TAX1BP1 not only promoted expressional upregulation of ND1, ND2, NDUFS2 and NDUFS4 in mitochondria, but also promoted their assemblies. Moreover, Knockdown of ND1 with siRNA significantly attenuated DPT-induced increases in complex I activity, mitochondrial superoxide generation and glioma cell death. Thus, TAX1BP1 promoted complex I activation by regulating the expression and assemblies of its subunits.

Although we did not investigate in the present study why TAX1BP1 could upregulate the expression and assemblies of the respiratory chain complex I, previous reports showed that multiple pathways might be involved in this process. It was found that TAX1BP1 could use its SKICH domain to interact with mitochondrial MTPAP, a RNA poly(A) polymerase responsible for maintaining the stability and maturation of tRNAs [36, 37] and its mutation could result in a severe loss of respiratory chain complex I and perturbation of de novo mitochondrial protein synthesis [38]. Thus, TAX1BP1 might regulate complex I activity via interaction with MTPAP. Additionally, PGC-1α whose expression is negatively regulated by NF-κB/p65 could also promote the expression of respiratory chain complex I subunits via its downstream NRF1 and TFAM [39, 40]. Notably, TAX1BP1 was a potent interior inhibitor for NF-κB/p65 [41], despite its underlying mechanism remains to be clarified. Thus, TAX1BP1 could also regulate complex I activity via upregulating PGC-1α expression. Furthermore, activated NF-κB is also involved in regulating the expressions of GPX4 and catalase, both of which are components of the mitochondrial anti-oxidation system. It was reported miR-93-5p inhibited the transcription of GPX4 in ovary granulosa cells by targeting NF-κB/p65 [42]. The downregulated expression of catalase induced by MLN4924 in human esophageal cancer cells was also related to inhibiting NF-κB/p65 [43]. In this study, we found TAX1BP1 knockdown also alleviated the downregulation of catalase and GPX4 caused by DPT. Thus, TAX1BP1 could also improve mitochondrial level of superoxide by decreasing the protein levels of GPX4 and catalase.

In addition to ionic radiation, ROS are crucial factors initiating DNA DSBs-dependent over-activation of PARP1, because nucleic acids are vulnerable to be attacked by ROS [44]. Moreover, it was found that hydrogen peroxide alone could cause DNA DSBs in glioma cells [45]. Consistently, previous studies showed that ROS contributed to shikonin- and TMZ-induced DNA DSBs in glioma cells [7, 15]. Furthermore, we found previously ROS contributed to DPT-induced glioma cell parthanatos [14]. In this study, inhibition of superoxide accumulation by rotenone or knockdown of ND1 with siRNA not only significantly attenuated DPT-induced improvement of ROS, but also suppressed DNA DSBs and PARP1 activation. Thus, the mitochondrial superoxide produced by activated respiratory chain complex I could reversely reinforce PARP1 activation by promoting ROS-dependent DNA DSBs.

In conclusion, we demonstrated that the NAD^+^ depletion caused by PARP1 overactivation resulted in mitochondrial translocation of TAX1BP1, which contributed to DPT-induced nuclear translocation of AIF and glioma cell parthanatos via upregulation of mitochondrial complex I activity and assembly.

## Supplementary information

Supplementary Figure

Supplementary figure legends
