# Supplementary figures and images for: TAX1BP1 contributes to deoxypodophyllotoxin-induced glioma cell parthanatos via inducing nuclear translocation of AIF by activation of mitochondrial respiratory chain complex I
Source: Acta Pharmacol Sin. 2023 Apr 25;44(9):1906–19. doi: 10.1038/s41401-023-01091-w (PMC10462642; doi:10.1038/s41401-023-01091-w)

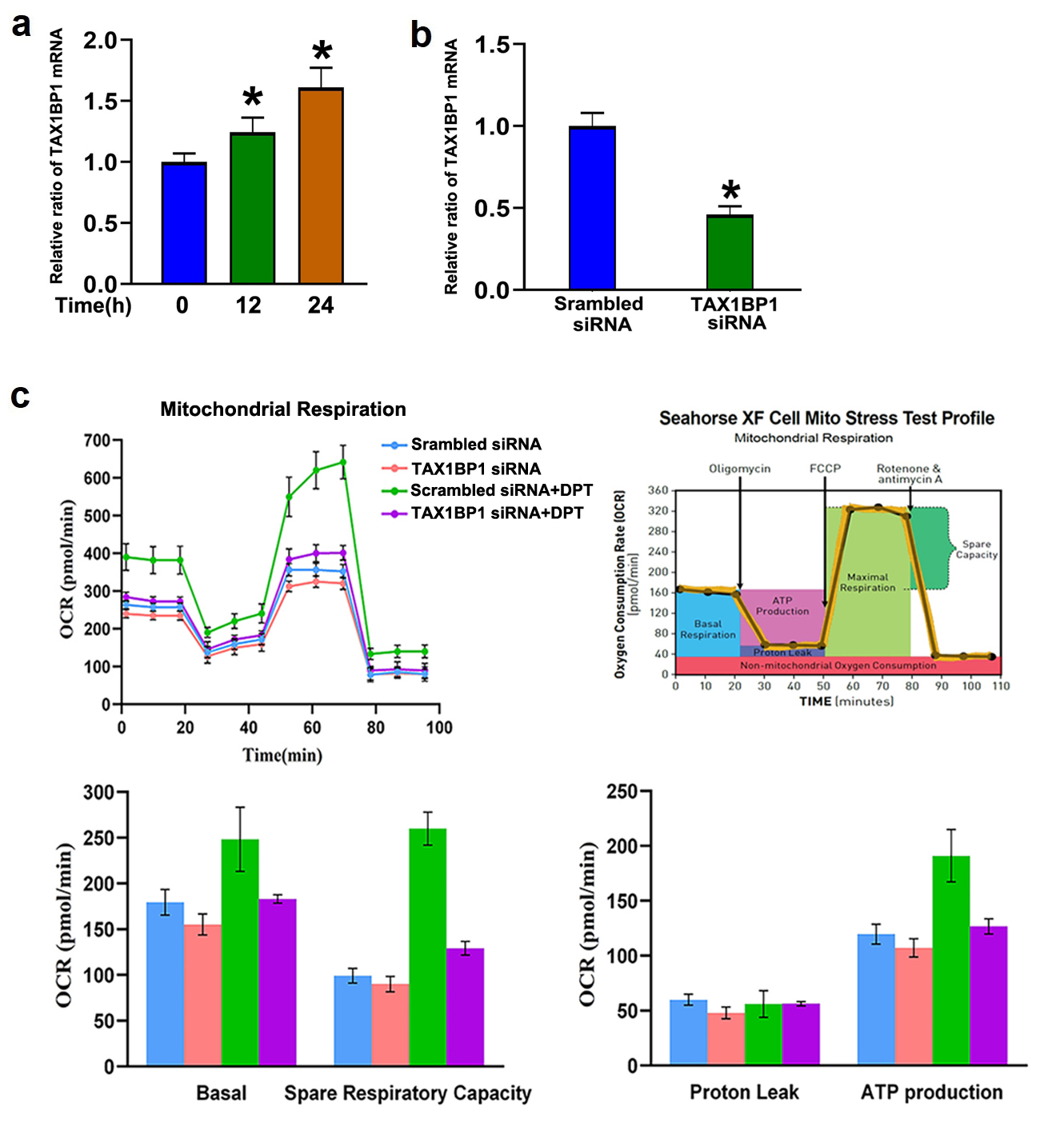

Supplement: Supplementary file 1 — Supplementary Figure [file 41401_2023_1091_MOESM1_ESM.tif]
