# Supplementary material for: TAX1BP1 contributes to deoxypodophyllotoxin-induced glioma cell parthanatos via inducing nuclear translocation of AIF by activation of mitochondrial respiratory chain complex I
Source: Acta Pharmacol Sin. 2023 Apr 25;44(9):1906–19. doi: 10.1038/s41401-023-01091-w (PMC10462642; doi:10.1038/s41401-023-01091-w)
Supplement: Supplementary file 2 — Supplementary figure legends [file 41401_2023_1091_MOESM2_ESM.docx]

Supplementary figure legends

(a) DPT induced time-dependent upregulation in mRNA of TAX1BP1 (TAX1BP1/β-Actin). (b) Efficiency of knockdown of TAX1BP1with siRNA (TAX1BP1/β-Actin). (c) DPT induced changes in mitochondrial stress.
